# Prevalence and risk factors of venous thromboembolism in postoperative patients: A retrospective study

**DOI:** 10.12669/pjms.346.16021

**Published:** 2018

**Authors:** Aylin Durmaz Edeer, Saadet Comez, Hale Turhan Damar, Aysegul savci

**Affiliations:** 1 *Dr. Aylin Durmaz Edeer, PhD. Department of Surgical Nursing, Dokuz Eylul University, Izmir, Turkey*; 2 *Dr. Saadet Comez, PhD. Department of Anesthesia, Mehmet Akif Ersoy University, Burdur, Turkey*; 3 *Dr. Hale Turhan Damar, PhD. Department of Surgical Nursing, Dokuz Eylul University, Izmir, Turkey*; 4 *Dr. Aysegul Savci, PhD. Department of Nursing, Health Science Faculty, Kutahya Health Science University, Kutahya, Turkey*

**Keywords:** Venous Thromboembolism, Postoperative, Patients, Prevalence

## Abstract

**Objective:**

To investigate the prevalence of and risk factors for Venous Thromboembolism (VTE) in postoperative patients.

**Methods:**

This descriptive, cross-sectional, retrospective study was conducted from August 2016 to October 2016 at two university hospitals and one public hospital. Total 217,354 patients records who underwent surgery in between 2010 and 2015 were examined. The study sample consisted of 123 patients who had postoperative venous thrombosis and pulmonary embolism, and whose discharge details, consultation data, diagnostic reports, and tests were examined in detail.

**Results:**

The prevalence of VTE in postoperative patients was 5.6/10,000. The mean age of the patients was 60.22±18.56 years. Of 123 patients, 51.20% were male, 30.90% were smokers, 46.30% had a comorbid disease, and 27.60% were diagnosed with cancer. Of the patients who had postoperative VTE, 65.0% had major surgery. Pharmacologic thromboprophylaxis was used in only 24.4% of patients (n=30).

**Conclusion:**

The prevalence of VTE in the present study is lower than that in other studies. Because surgery is a risk factor for VTE, patients who will be operated should be assessed. Considering the present results, we can assume that patients’ conditions are not being assessed appropriately. In addition, findings indicate that a standard for preventing VTE has not yet been established.

## INTRODUCTION

Venous Thromboembolism (VTE) includes both Deep Venous Thrombosis (DVT) and Pulmonary Embolism (PE); it is the third most common cardiovascular disease after myocardial infarction and stroke.^1^ In United States of America, approximately 900,000 people experience VTE every year. In addition, approximately 100,000 people die due to unknown reasons related to VTE.^2^ VTE is a serious but preventable health issue.

There are three factors that play a role in the development of VTE: damages to the venous endothelium, venous stasis, and hypercoagulability.^3^ There is another factor that plays a role in the development of VTE, and this factor is surgery.^3^–^6^ Venous stasis may develop in postoperative patients due to physical inactivity and fewer skeletal muscle contractions. In addition, the venous endothelium may be damaged by surgical procedures or trauma. Surgical trauma reduces antithrombin III level and causes pressure on fibrinolytic activity, thus hypercoagulability may develop.^3^ Therefore, some postoperative patient groups are at risk of VTE development. In a multicenter study, 68,183 patients at 358 hospitals in 32 countries were examined for risk factors related to VTE. Of the patients hospitalized for all reasons, 51.8% face VTE risk. However, the same risk threatens 64.4% of the surgical patients.^7^ In another study, this risk involved 53.6% of the hospitalized patients and 61.3% of the surgical patients.^8^ In a multicenter study conducted in Turkey, 1701 patients at 11 hospitals were examined for VTE risk: this study demonstrated that 35.6% of all patients and 64.9% of the surgical patients face VTE risk.^9^ Another study that examined the VTE risk for the patients at the surgical clinics in Turkey showed that 62.1% of these patients have a high VTE risk.^10^ The risk of developing VTE is very high for patients who undergo surgical operation, but only a limited number of the studies examine postoperative VTE incidence in Turkey. In one study examining the VTE incidence following the vascular surgery in Turkey, the VTE incidence was found to be 1.75%.^11^ Studies related to VTE incidence following various operations were found in USA and in Asian countries, for example, Singapore and India;^12^-^19^ the VTE incidence rate ranged between 5/10,000 and 13% in these studies. In two studies conducted in Australia and Spain, postoperative VTE prevalence was found to be 1.8-2‰.^20^,^21^ VTE causes pulmonary embolism, which accounts for the vast majority of hospital deaths. VTE causing death can also be prevented at the same time.^1^,^2^,^19^ However, we were unable to find a summary examining postoperative VTE prevalence in all Turkey. Detecting VTE prevalence and effective risk factors for postoperative patients is important for creating care protocols that professionals in this field can follow. Thus, the present study was conducted to examine prevalence of VTE and risk factors for the patients who underwent operations at university and public hospitals in Izmir, Turkey.

## METHODS

This is a retrospective and descriptive study. It was conducted at university and public hospitals in Izmir, Turkey. Data were collected between August 2016 and October 2016. The study population included patients who underwent a surgery at two university hospitals and one public hospitals in Izmır between 2010 and 2015. The plan was to get in contact with all these patients. Failure to keep the patient records of a university hospital in the electronic media, irregular patient records, and issues in reading these records were among the exclusion criteria. In addition, 24 public hospitals with no sufficient and regular data record, despite having an electronic patient database, were excluded. After exclusions, we contacted. Total 217,354 patients who underwent a surgery in two university hospitals and one public hospital.

**Fig.1:**
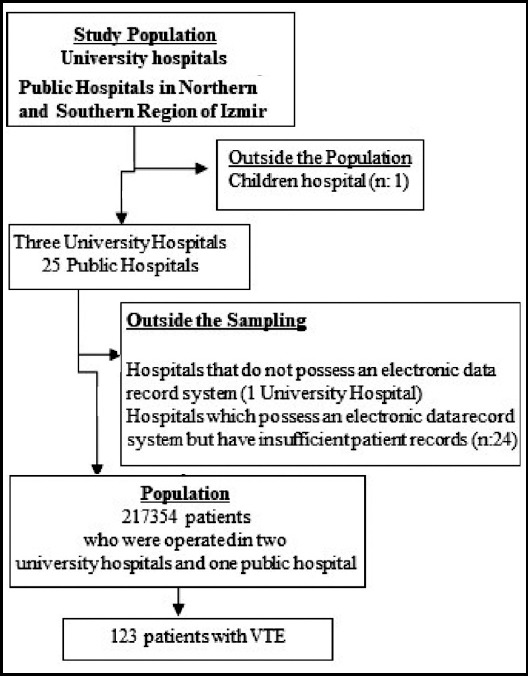
Study population diagram.

The patient sample included 123 patients over 18 years of age who had operations between 2010 and 2015 and experienced DVT and PE (Fig.1). Patients who were hospitalized upon the DVT and PE diagnosis but had not undergone surgery were excluded.

### Data Collection

Data were collected using the “Hospital Information System” at university and public hospitals in Izmir. International Classification of Diseases (ICD-10) is used within this system. “Venous thromboembolism” and “pulmonary embolism” diagnoses were examined based on this classification. Discharge details, consultation reports, and diagnostic methods and tests of the patients who underwent surgery between 2010 and 2015 were analyzed in detail. Patients who were diagnosed with DVT and PE before undergoing surgery were excluded from the sampling; this left a study sample of 123 patients who had VTE following surgery. Data related to risk factors were collected from patient records using the same system in combination with the VTE risk factor detection form constructed by the present researchers based on VTE literature. This form consists of 18 questions regarding age, sex, smoking, operation type, duration of hospital stay, VTE history, cancer history, and anticoagulant use.^1^,^7^,^22^ Permission was obtained from the participating institutions and ethics committees.

### Evaluating the Data

The data were analyzed using SPSS 15.0. Descriptive statistical data such as numbers, percentage, and means were used to analyze patient details

## RESULTS

The mean age of the patients who were operated and developed VTE was 60.22 ± 18.56 (range, 20 to 94 years). Of the patients, 52.80% were older than 60 years, and 51.20% were male. Smokers comprised 30.90% of the patient sample, and 46.30% had a comorbid disease. Of the patients who had VTE following the operations, 65.0% had a major surgery: 29.30%, orthopedic surgery; 25.20%, general surgery; and 19.50%, cardiovascular surgery. Of the patients who had postoperative VTE, 27.60% were diagnosed with cancer (Table-I). VTE prevalence among the operated patients was found to be 5.6/10,000.

**Table-I:** Sociodemographic and clinical characters of the patients who developed VTE in surgical patients.

Patients’ Characteristics	(X±SD)	
Mean Age	60.22±18.56	
Duration of Hospital Stay	9.76±11.94	

	n	%

***Sex***	
Female	60	48.8
Male	63	51.2
***Comorbid Disease***	
Yes	57	46.3
No	66	53.7
***Smoking***	
Yes	38	30.9
No	85	69.1
***Operation Type***	
Orthopedic	36	29.3
General (abdomen surgery)	31	25.2
Cardiovascular	24	19.5
Otorhinolaryngology	10	8.1
Gynecology and Obstetrics	7	5.7
Urology	6	4.9
Brain and Nerve	6	4.9
Other (Thoracic and Plastic)	3	2.4
***Surgery Type***	
Major	80	65.0
Minor	43	35.0
***Cancer***	
Yes	34	27.6
No	89	72.4
***VTE history***	
Yes	5	4.1
No	118	95.9
***Thromboprophylaxis***	
Yes	30	24.4
No	93	75.6

Pharmacologic thromboprophylaxis was used in only 24.4% of patients (n = 30). Of the patients who received pharmacologic thromboprophylaxis (n = 23), 76.7% had major operations (n = 16), and 53.3% had orthopedic surgery. Only 16.7% of the patients who received pharmacologic thromboprophylaxis (n = 5) were diagnosed with cancer.

In the present study, 45 patients over the age of 60 years had major operations. Patients who developed VTE constituted 36.5%; 42.2% of the patients who were over 60 and had major operations received pharmacologic thromboprophylaxis (n = 19), and 57.8% of the patients who were at high risk of VTE did not receive pharmacologic thromboprophylaxis.

The present study includes 34 patients who were diagnosed with cancer or had recovered from cancer. Of these 34 patients, 58.80% were over 60 (n = 20), and 76.50% had major operations (n = 26). The number of patients who were over 60, diagnosed with cancer or had recovered from cancer, and had a major surgery was 12; these patients constituted 9.75% of the patients with VTE. Pharmacologic thromboprophylaxis was used in only 16.66% of this group (n = 2). Eighteen patients over 60 years of age had femur fracture surgery and elective femur prosthesis. Patients who developed VTE constituted 14.6% of that patient group. Pharmacologic thromboprophy laxis was used in 66.66% of those patients over 60 who had a femur fracture operation and elective femur prosthesis (n = 12). No data regarding the methods (mechanical prophylaxis, pressurized elastic stocking, intermittent pneumatic compression) other than pharmacologic thromboprophylaxis that were used in the patients who were operated were available.

## DISCUSSION

VTE may develop in postoperative patients for various reasons: patient characteristics (advanced age, comorbid disease, for example), inactivity following the operation, and insufficient venous return. VTE prevalence among the postoperative patients was 5.6/10,000 in the present study. In a study that examined the records of 4,223,317 patients operated at 86 hospitals in Australia between 2002 and 2009, VTE prevalence was 2/1000.^20^ In addition, VTE prevalence among 6004 patients who underwent chest surgery in Spain between 1994 and 2011 was 1.8%.^21^ VTE prevalence in the present study is lower than that in other studies. The greatest limitation in this study is the insufficient amount of information provided about the patients in hospital information system. We excluded some data due to insufficient information regarding when and why VTE developed in the patients. For example, when a patient visited an institution that was included in the sampling for his/her VTE complaints although his/her operation was not performed in a hospital that was not included in the sampling, healthcare staff might have overlooked the fact that VTE might have developed following the operation or findings might have been recorded differently. Thus, the VTE prevalence found in the present study may be lower than that of other studies.

A surgical operation is a risk factor for VTE: therefore, patients to be operated should be evaluated taking this into consideration. Various treatment guidelines for preventing VTE have been published.^23^-^26^ The Turkish Thoracic Society recommends, in “The Report for Pulmonary Thromboembolism Diagnosis and Treatment Consensus”, that patients should be evaluated considering the type, duration, and area of surgery, and additional clinical risks that the surgery poses for the patient. The patient’s age (> 60 years), presence of cancer, major surgery (> 45 min) history, femur fracture surgery, or prosthesis application are among the factors that increase VTE risk, and when these factors are present, pharmacologic thromboprophylaxis should be applied based on the consensus report. In the present study, 42.2% of the patients over 60 years of age and had major operations received pharmacologic thromboprophylaxis. Patients who were over 60 and had the risk factor of a history of major surgery were included in the high-risk group. In a study conducted in Singapore, 10.2% of the patients who had major operations received thromboprophylaxis for VTE. Another study in India indicated that 16.3% of the operated patient group received thromboprophylaxis. Of the patients in a study conducted in England, 37% received thromboprophylaxis.^8^,^19^ The multicenter study conducted VTE risk and prophylaxis use among operated and internal disease patients in 32 countries; prophylaxis was used in 58.5% of the patients who were operated and had VTE risk.^7^

The study conducted by Ongen et al. with 1701 patients at 11 hospitals in Turkey indicated that prophylaxis was used in 39% of the operated patients exhibiting the risks.^9^ In the study conducted by Kurtoglu et al. with 1472 patients at 20 different general surgery clinics, 66.9% of the general surgery patients received prophylaxis for VTE.^10^ The pharmacologic thromboprophylaxis application rate was found to be low in some studies, but higher in two studies when compared with the present study. A low pharmacologic thromboprophylaxis application rate may be because doctors do not want to face the risk of hemorrhage that is the most significant adverse effect of pharmacologic thromboprophylaxis medicines administered following an operation.

A high pharmacologic thromboprophylaxis application rate indicates that patients’ risks were evaluated. Almost half of all patients in the present study received pharmacologic thromboprophylaxis, which indicates that doctors approach with caution in view of its known adverse effects. In addition, 16.66% of the patients over 60, diagnosed with cancer, or in recovery from cancers and having had a major surgery received pharmacologic thromboprophylaxis. VTE risk becomes higher for patients who have all these risk factors. In the study conducted by Clayburgh et al., 14% of the patients who have head and neck cancers received anticoagulant following the operation.^12^ The outcome of the Clayburgh study resembles that of our study. Failure to use diagnosis tools for VTE in surgery clinics during the preoperative process may constitute an obstacle for determining the risky situation for the patients. Pharmacologic thromboprophylaxis was applied to 66.66% of those who were over 60 and had had a femur fracture operation and elective femur prosthesis. The pharmacologic thromboprophylaxis application rate was found to be higher among the orthopedic surgery patients when compared with other patient groups. The multicenter study conducted by Cohen et al. indicated that 88.8% of the patients who were treated with femur and knee prosthesis received pharmacologic thromboprophylaxis.^7^ In the study conducted by Ongen et al., pharmacologic thromboprophylaxis was applied to 83.3% of the patients who underwent surgery for femur fracture, and 66.7% of those who had femur prosthesis operation.^9^ Studies have suggested that the pharmacologic thromboprophylaxis application rate is high among the patients who will have femur fracture and prosthesis operation. This rate is low in our study by comparison. Orthopedic operations are risky for VTE. Particularly, patients who will undergo femur fracture and prosthesis operations are included in the patient group that has risks for VTE.^7^ It is important to plan to include pharmacologic thromboprophylaxis in the treatment. Of the patients who had major orthopedic operations and received VTE prophylaxis, 1.3% had VTE. Due to the necessary extended immobilization, VTE is a significant complication. The pharmacologic thromboprophylaxis application rate may be high because orthopedic surgeons consider this issue. However, one-third of the patients in our study did not receive pharmacologic thromboprophylaxis, indicating that this problem is not negligible and that a standard has not yet been established for preventing VTE.

No data were found regarding mechanical prophylaxis use, such as pressurized elastic stocking or intermittent pneumatic compression for the patients who were operated, although studies have demonstrated that these methods were effective. Using these methods for the patients for whom the hemorrhage risk is present is important for preventing VTE development. These methods should be considered while planning the treatment.

## CONCLUSIONS

Because VTE is a lethal but preventable disease, caregivers should carefully evaluate the risk for the individual patient before the operations. Using an easy-to-use and understandable VTE risk evaluation tool for the surgery in the preoperative process will help plan the most appropriate treatment and form a common language. Prophylaxis against VTE is cost effective for many surgical patients and should be implemented in all clinical settings where its effectiveness and safety has been established.

### Authors Contribution

**ADE:** Conceived, designed and did statistical analysis & editing of manuscript.

**ADE, SC, HTD & AS:** Did data collection and manuscript writing.

**ADE, SC, HTD & AS:** Did review and final approval of manuscript.
